# Severe Symptomatic Primary CMV Infection in Inflammatory Bowel Disease Patients with Low Population Seroprevalence

**DOI:** 10.1155/2018/1029401

**Published:** 2018-06-28

**Authors:** Catherine Rowan, Ciaran Judge, Mary D. Cannon, Garret Cullen, Hugh E. Mulcahy, Elizabeth Ryan, Cillian F. De Gascun, Glen A. Doherty

**Affiliations:** ^1^Centre for Colorectal Disease, St. Vincent's University Hospital, Dublin, Ireland; ^2^National Virus Reference Laboratory, University College Dublin, Dublin, Ireland

## Abstract

**Background:**

Cytomegalovirus disease in patients with inflammatory bowel disease is frequently the result of viral reactivation. Conversely, primary CMV infection is believed to be uncommon in immunocompetent adults due to high population seroprevalence.

**Objectives:**

The aim of this study was to examine the frequency and severity of primary cytomegalovirus infection in an adult cohort of IBD patients.

**Study Design:**

A retrospective review of a prospectively maintained database of 3200 IBD patients attending a single academic centre was performed. Patients with primary CMV infection 2010–13 were identified; clinical, serologic, and virologic parameters were studied in detail. The seroprevalence of CMV in the patient population was also evaluated.

**Results:**

Eight patients with IBD (UC = 3, IBD-U = 1, CD = 4) presented with primary CMV infection. Patients presented with both gastrointestinal and extraintestinal symptoms. Mean age was 33 years, and median duration of disease was 72 months. All eight patients were receiving a thiopurine immunomodulator. Median duration of IM use was 144 weeks (range 7–720 weeks). All 8 patients required hospitalisation, with 1 ICU admission; the median length of hospital stay was 11 days (range 6–27). Infection resolved in all cases with withdrawal of immunomodulator and/or antiviral therapy. Seroprevalence of IgG to CMV, indicating prior exposure, in a subgroup of IBD patients (*n* = 80) was 30.5% and increased with age.

**Conclusion:**

Primary cytomegalovirus infection can cause a severe illness in IBD patients, particularly those receiving immunosuppression. In areas where adult CMV seroprevalence is low, evidence of CMV should be sought in IBD patients presenting with any febrile systemic illness.

## 1. Background

Cytomegalovirus (CMV) is a double-stranded, enveloped DNA virus from the Herpesviridae family. This virus shares the defining characteristic of the *Herpesvirus* family, in its capacity to establish latency in the host following primary infection. As such, CMV disease can be the consequence of both primary infection and reactivation, manifesting in colonic tissue or a range of extraintestinal locations, for example, hepatitis, retinitis, and pneumonitis.

CMV is a ubiquitous virus with the seroprevalence in the general population ranging from 40 to 100% [1]. This prevalence varies geographically, however, and by socioeconomic group, with higher seroprevalence rates reported in less industrialised countries and in populations with lower socioeconomic status. Of note, the seroprevalence of CMV in adults in Ireland is low by international standards.

Primary CMV infection in the immunocompetent host is often asymptomatic or manifests as a mild, self-limiting, febrile, mononucleosis-type illness. In contrast, primary CMV infection in the immunocompromised individual can be associated with considerable morbidity and mortality [2]. Congenital cytomegalovirus (CMV) infection is the leading nongenetic cause of sensorineural hearing loss (SNHL) and is the most frequent known cause of mental retardation [3, 4].

The role of CMV in inflammatory bowel disease (IBD) has been a constant topic of interest since the first case was described in 1961 [5, 6]. IBD patients can be considered at increased risk of CMV, both primary and reactivation, for a number of reasons. Immune dysregulation, reduced barrier function, malnutrition, and the use of immunomodulators such as azathioprine all contribute to the immunosuppressed state of many IBD patients. The prevalence of CMV reactivation in moderate-to-severe colitis has been quoted between 21% and 34% [7], and prevalence of CMV in steroid-refractory colitis is reported as greater than 30% [8].

Little is known about primary CMV in IBD patients, and there is conflicting evidence as to whether CMV actually affects the course of disease. Although current European Crohn's and Colitis Organisation (ECCO) guidelines advise the discontinuation of immunomodulators and initiation of antiviral treatment in the setting of CMV detected in patients with acute severe colitis, the guidelines do not distinguish between primary CMV and reactivation [9].

## 2. Objectives

Thus, the aim of this study was to examine the frequency and severity of primary CMV-related disease in a cohort of adult IBD patients.

## 3. Study Design

A retrospective review of a prospectively maintained database of 3200 IBD patients attending a single academic centre was performed. Patients diagnosed with CMV disease between 2010 and 2013 were identified. Diagnosis of primary CMV was based on serological and virological parameters, that is, CMV DNA in plasma, detectable CMV IgM, and the presence of low avidity IgG in the acute setting. IgG avidity is defined as the strength with which IgG binds to antigenic epitopes expressed by a given protein. IgG avidity assays measure antibody maturity. They have been shown to reliably detect recent primary CMV infection. When a person is infected with CMV for the first time, the body produces low-avidity IgG, which matures over subsequent months. Low CMV IgG avidity is an accurate indicator of primary infection within the preceding 2–4 months, whereas high-avidity CMV IgG indicates past infection.

Demographic data were obtained, including age, sex, IBD type, and duration. Organ involvement, symptoms, treatment (immunomodulators, anti-TNF, etc.) at the time of diagnosis, and use of antiviral therapy subsequent to diagnosis were extracted from the IBD patient database and from chart review. White cell count, haemoglobin, liver function tests, serology, and pathological data were included from the laboratory records. Outcome measures assessed were the need for surgical intervention within 12 months of CMV infection, length of stay, and mortality.

Seroprevalence of CMV was also assessed using a baseline infection screen performed in asymptomatic patients starting immunomodulator or anti-TNF therapy, between 2010 and 2013.

Initial CMV-specific IgM and IgG serology was performed on the Abbott Architect analyser; CMV IgM confirmation and CMV IgG avidity testing were performed on the BioMérieux VIDAS platform; CMA DNA levels were measured using the Qiagen artus kit; all assays were carried out in accordance with the manufacturer's instructions.

### 3.1. Statistics

Continuous variables were described using standard descriptive statistics such as mean, median, and range. The chi-squared test was used to assess for statistically significant differences in seroprevalence between groups. A *p* value of <0.05 was deemed to be significant, with confidence intervals of 95%.

## 4. Results

### 4.1. Characteristics of Primary CMV Infection

Eight patients (6 female) with primary clinical CMV infection were identified; clinical, serologic, and virologic parameters were studied in detail. One patient did not have positive CMV IgM but had high titres of CMV DNA and previous negative CMV serology. Six of the 8 cases described had CMV DNA titres recorded in addition to their serology. There was a total of 9315 patient years of follow-up for this IBD cohort. Amongst patients exposed to an immunomodulator, the incidence of primary CMV infection was 0.02 cases/patient year. Primary CMV infection was defined serologically as the presence of CMV-specific IgM in conjunction with low-avidity IgG. Two of the 8 patients described had negative CMV IgG documented prior to admission, corroborating the above serology.

Of the eight patients with IBD (ulcerative colitis = 3, IBD-undetermined = 1, Crohn's disease = 4) who presented with primary CMV infection, 4 had evidence of CMV colitis. CMV was confirmed histologically in colonic biopsies from 3 patients. Patients who did not present with symptoms of colitis did not undergo endoscopic evaluation. Of those patients who had CMV colitis, 2 patients had Crohn's disease with colonic involvement. The 2 remaining patients with UC were shown to have extracolonic CMV disease in addition to CMV colitis. This included CMV duodenitis and gastritis demonstrated histologically in biopsies taken from 1 patient. Another patient with UC developed respiratory compromise during the hospital admission and was diagnosed with CMV pneumonitis. Patient demographics and disease distribution are described below in Table 1.

Median CMV DNA levels were 23,022 IU/ml (IQR 9532.8–31,330.2 IU/ml). All patients required hospitalisation (ICU admission, *n* = 1); the median length of hospital stay for the cohort was 11 days (range 6–27), with no statistically significant difference for the subgroup of patients with colonic involvement (*p* = 0.41).

**Figure 1:**
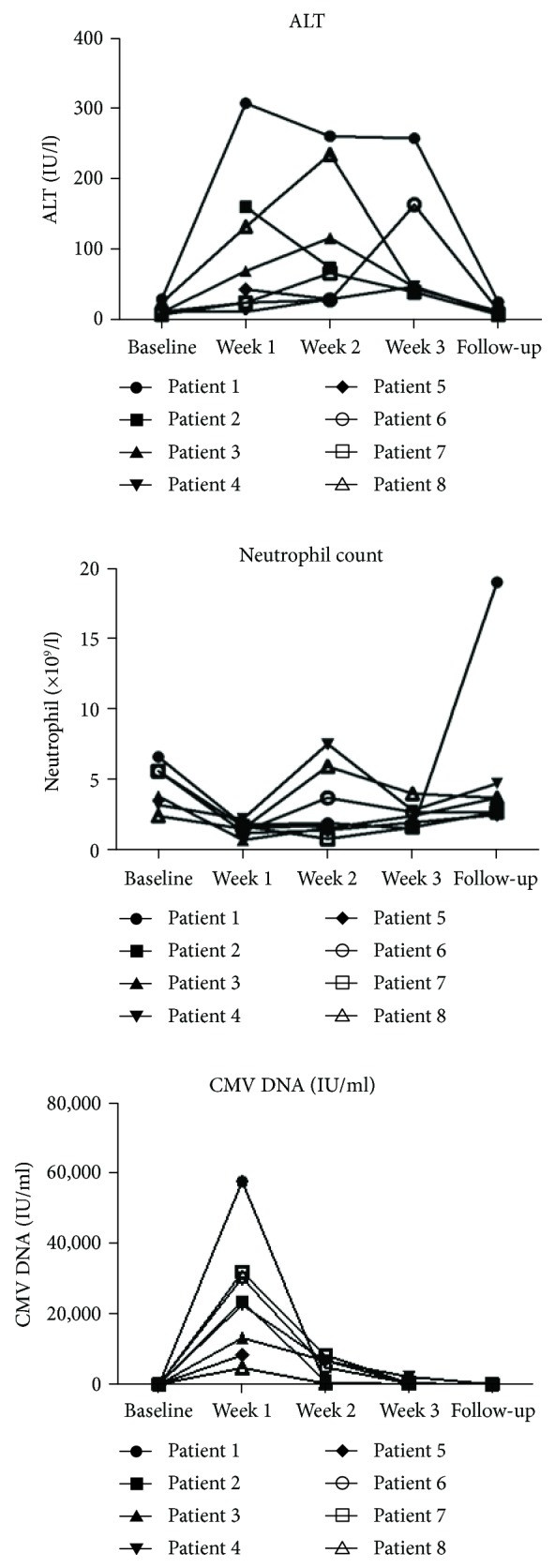
Trend in alanine transferase (ALT) (IU/ml), neutrophil count (×10^9^/l) and CMV DNA (IU/ml) over time from baseline to time of the last follow-up.

All patients were receiving a thiopurine immunomodulator (IM) at the onset of CMV infection (attributable risk 100%). Two patients were receiving a combination of azathioprine and an anti-TNF agent at the time of diagnosis. The median duration of IM use was 144 weeks (range 7–624 weeks). Seven patients (87.5%) were neutropenic at the time of admission and also had abnormal liver function test (Figure 1). However, no patients had preexisting neutropenia or deranged liver function tests prior to hospitalisation that would have indicated drug toxicity.

CMV infection resolved in all cases. In 6 cases, withdrawal of IM with instigation of ganciclovir/valganciclovir therapy was necessary. One patient was treated with ganciclovir only, while 1 patient received a 3-week course of valganciclovir alone. The remaining 4 patients received intravenous ganciclovir 5 mg/kg bd followed by a course of valganciclovir. The mean duration of treatment with ganciclovir was 10 days (range 5–27 days) while the mean duration of treatment with valganciclovir was 12 days (range 6–21 days). The development of adverse side effects, including encephalopathy, prompted withdrawal of ganciclovir after 5 days in one case. These symptoms resolved following cessation of antiviral therapy. In the remaining 2 patients (CMV hepatitis; CMV respiratory infection), the IM was withdrawn but no antiviral treatment was given.

In 4 instances, the IM was subsequently restarted once clinicians were satisfied of full resolution of CMV infection. The median time to restarting azathioprine was 69 days (IQR 32.5–92 days). Three patients had sequential monitoring of plasma CMV DNA levels, and azathioprine was reinstituted once CMV DNA was undetectable. One patient restarted immunomodulator therapy based on clinical progress. All four patients had continued azathioprine therapy at the time of the last follow-up and were clinically well.

There were no deaths recorded, and none of the 8 patients proceeded to surgery in the 12 months following CMV infection.

### 4.2. Seroprevalence of CMV in IBD Population

Seroprevalence for CMV, Epstein Barr virus (EBV), and varicella zoster virus (VZV) was assessed in patients with IBD who had baseline infection screens performed prior to immunosuppression from 2010 to 2013 (*n* = 376). This included patients screened prior to IM and biologic therapy. In our centre, CMV and EBV are not part of a standard preimmunosuppression screen so there was variation in the serology requested for each patient. The presence of a positive CMV IgG, EBV IgG, or VZV IgG was viewed as indicating prior exposure to the viruses. Of those patients screened for CMV (*n* = 80), 24 (30%) were seropositive. CMV seroprevalence increased with age. For example, in those patients over 60 years of age, seroprevalence was calculated at 50%, compared to 25.75% in the under 60 yrs cohort (*p* = 0.07).

The seroprevalence rates of VZV and EBV were calculated and were significantly higher than that of CMV (Table 2).

## 5. Discussion

This case series highlights the significant morbidity associated with primary CMV infection in adult patients with IBD who are taking thiopurine immunomodulators.

The seroprevalence of CMV in adults of reproductive age shows considerable geographic variation, typically ranging from 40 to 100% [10]. The seroprevalence is lowest in Western Europe and the USA, although within these countries, seroprevalence may be 20–30% higher in nonwhites and is associated with lower socioeconomic status. CMV seroprevalence in Ireland is considerably lower when compared to other industrialised countries such as Australia (57%) [11], USA (50.4%) [12], and Portugal (77%) [13]. Indeed, the seroprevalence of CMV amongst Irish-born women engaged in antenatal care has been reported to be amongst the lowest in the world [14] (30%, compared to almost 90% in women tested in Ireland but born elsewhere). In addition, the CMV seroprevalence in Irish solid organ transplant donors was 33.4% (range 22–48% per annum) over the time period 1990–2013 [15]. Consequently, the seroprevalence of CMV reported in our IBD cohort, estimated at 30%, is in keeping with the data from previous Irish studies. This relatively low seroprevalence may explain why primary CMV infection occurs more commonly in adults in this population.

Primary CMV infection in immunocompetent adults is typically asymptomatic; however, it can also cause a mild systemic illness, with typical features of mononucleosis, and rarely requires hospital treatment. However, this study highlights that in IBD patients, particularly those receiving thiopurine immunomodulators, CMV infection can present in a multitude of ways. The patients described in this case series presented with CMV affecting a range of extraintestinal sites as well as CMV colitis, which resulted in prolonged hospital admission and in one instance necessitated transfer to ICU. In contrast to CMV reactivation in the setting of acute severe ulcerative colitis, half of the patients presenting with CMV colitis also demonstrated extracolonic disease, such as CMV pneumonitis.

Furthermore, as per consensus guidelines, immunomodulators should be withdrawn and in many cases are not reinstituted. Azathioprine and 6-mercaptopurine are commonly used alone and in combination with anti-TNF therapy. The SONIC trial demonstrated a statistically significant difference in steroid-free remission with the use of combination therapy compared with azathioprine or infliximab alone [16]. The use of combination therapy is becoming more common in clinical practice, often as a way to augment treatment effect and limit antibody formation. A single patient with CMV colitis in this case series was on anti-TNF at the time of diagnosis. This was discontinued when antiviral therapy was commenced and not restarted. A recent retrospective study did not find any significant difference in colectomy rates amongst patients treated with anti-TNF and antiviral compared to antiviral therapy alone [17]. However, it is unclear if and when immunomodulators or combinations therapy can be prescribed following CMV infection, and only half of our patients restarted azathioprine therapy.

This poses challenges for the clinician; although immunomodulators remain a vital component in the medical armoury against IBD, colitis can be a presenting symptom for both primary CMV or an exacerbation of the patient's underlying IBD. As such, the authors believe that it is important to exclude primary CMV infection (and indeed any infectious pathogen) before increasing immunosuppression in the acute setting. Any subsequent decision regarding treatment options, for example, escalation to combination therapy, will be tempered by a history of CMV infection. Consequently, this may result in limited therapeutic choices for patients with a complex, chronic inflammatory disease.

There are some limitations to this study, primarily the retrospective nature and the small patient numbers. Nonetheless, this is, to our knowledge, the first review of primary CMV infection in the IBD population to date, and the authors believe that this topic will become increasingly relevant in the context of falling CMV seroprevalence [18] in developed countries and the use of immunomodulators, particularly in combination with monoclonal antibodies as maintenance therapy in IBD.

## Figures and Tables

**(a):** 

Age (median)	33 years (24–44 yrs)
Gender (F : M)	6 : 2
Disease duration (median)	66 months (7–204)

**(b):** 

Disease distribution			
UC/IBD-U	*N* = 4	Crohn's disease	*N* = 4
Proctitis	1	A1	0
Left sided	3	A2	4
Pancolitis	0	A3	0
		L1	2
		L2	1
		L3	1
		L4	0
		B1	1
		B2	1
		B3	2
		P	2
*Surgery*	0		
*Medical treatment*			
Immunomodulators	4		4
5ASA	4		2
Anti-TNF	1		2
Steroid	2		1

**Table 2:** VZV, EBV, and CMV seroprevalence.

	VZV (*n* = 129)	EBV (*n* = 74)	CMV (*n* = 80)	
Seroprevalence	97.6% (*n* = 126)	86.5% (*n* = 64)	30% (*n* = 24)	*p* = 0.00001
<60 years	98% (*n* = 109)	84% (*n* = 54)	25.75% (*n* = 17)	
>60 years	94% (*n* = 17)	100% (*n* = 10)	50% (*n* = 7)	

## Data Availability

Data pertaining to the article is archived in the Centre for Colorectal Disease for review if necessary.

## References

[1] Al-Zafiri R., Gologan A., Galiatsatos P., Szilagyi A. (2012). Cytomegalovirus complicating inflammatory bowel disease: a 10-year experience in a community-based, university-affiliated hospital. *Gastroenterology & Hepatology*.

[2] Bruminhent J., Razonable R. R. (2014). Management of cytomegalovirus infection and disease in liver transplant recipients. *World Journal of Hepatology*.

[3] Kimberlin D. W., Jester P. M., Sánchez P. J. (2015). Valganciclovir for symptomatic congenital cytomegalovirus disease. *New England Journal of Medicine*.

[4] Jückstock J., Rothenburger M., Friese K., Traunmüller F. (2015). Passive immunization against congenital cytomegalovirus infection: current state of knowledge. *Pharmacology*.

[5] Powell R. D., Warner N. E., Levine R. S., Kirsner J. B. (1961). Cytomegalic inclusion disease and ulcerative colitis: report of a case in a young adult. *The American Journal of Medicine*.

[6] Lawlor G., Moss A. C. (2010). Cytomegalovirus in inflammatory bowel disease: pathogen or innocent bystander?. *Inflammatory Bowel Diseases*.

[7] Delvincourt M., Lopez A., Pillet S. (2014). The impact of cytomegalovirus reactivation and its treatment on the course of inflammatory bowel disease. *Alimentary Pharmacology & Therapeutics*.

[8] Ayre K., Warren B. F., Jeffery K., Travis S. P. L. (2009). The role of CMV in steroid-resistant ulcerative colitis: a systematic review. *Journal of Crohn's and Colitis*.

[9] Rahier J. F., Magro F., Abreu C. (2014). Second European evidence-based consensus on the prevention, diagnosis and management of opportunistic infections in inflammatory bowel disease. *Journal of Crohn's and Colitis*.

[10] Cannon M. J., Schmid D. S., Hyde T. B. (2010). Review of cytomegalovirus seroprevalence and demographic characteristics associated with infection. *Reviews in Medical Virology*.

[11] Seale H., MacIntyre C. R., Gidding H. F., Backhouse J. L., Dwyer D. E., Gilbert L. (2006). National serosurvey of cytomegalovirus in Australia. *Clinical and Vaccine Immunology*.

[12] Bate S. L., Dollard S. C., Cannon M. J. (2010). Cytomegalovirus seroprevalence in the United States: the National Health and Nutrition Examination Surveys, 1988–2004. *Clinical Infectious Diseases*.

[13] Lopo S., Vinagre E., Palminha P., Paixao M. T., Nogueira P., Freitas M. G. (2011). Seroprevalence to cytomegalovirus in the Portuguese population, 2002–2003. *Euro Surveillance*.

[14] Knowles S. J., Grundy K., Cahill I., Cafferkey M. T., Geary M. (2005). Low cytomegalovirus sero-prevalence in Irish pregnant women. *Irish Medical Journal*.

[15] Hassan J., O’Neill D., Honari B. (2016). Cytomegalovirus infection in Ireland; seroprevalence, HLA class I alleles, and implications. *Medicine*.

[16] Colombel J. F., Sandborn W. J., Reinisch W. (2010). Infliximab, azathioprine or combination therapy for Crohn’s disease. *The New England Journal of Medicine*.

[17] Kopylov U., Papamichael K., Katsanos K. (2017). Impact of infliximab and cyclosporine on the risk of colectomy in hospitalized patients with ulcerative colitis complicated by cytomegalovirus—a multicenter retrospective study. *Inflammatory Bowel Diseases*.

[18] de Ory F., Ramírez R., García Comas L., León P., Sagües M. J., Sanz J. C. (2004). Is there a change in cytomegalovirus seroepidemiology in Spain?. *European Journal of Epidemiology*.

